# In Situ Observation of Electron-Beam-Induced Formation of Nano-Structures in PbTe

**DOI:** 10.3390/nano11010163

**Published:** 2021-01-10

**Authors:** Iryna Zelenina, Igor Veremchuk, Yuri Grin, Paul Simon

**Affiliations:** 1 Max-Planck-Institut für Chemische Physik Fester Stoffe, Nöthnitzer Str. 40, 01187 Dresden, Germany; Iryna.Zelenina@cpfs.mpg.de (I.Z.); i.veremchuk@hzdr.de (I.V.); grin@cpfs.mpg.de (Y.G.); 2 Helmholtz-Zentrum Dresden-Rossendorf, Bautzner Landstr. 400, 01314 Dresden, Germany

**Keywords:** lead telluride, PbTe, thermoelectric material, in situ, TEM, nano-bar, nano-cube, nano-layer

## Abstract

Nano-scaled thermoelectric materials attract significant interest due to their improved physical properties as compared to bulk materials. Well-shaped nanoparticles such as nano-bars and nano-cubes were observed in the known thermoelectric material PbTe. Their extended two-dimensional nano-layer arrangements form directly in situ through electron-beam treatment in the transmission electron microscope. The experiments show the atomistic depletion mechanism of the initial crystal and the recrystallization of PbTe nanoparticles out of the microparticles due to the local atomic-scale transport via the gas phase beyond a threshold current density of the beam.

## 1. Introduction

Nanoparticles are the focus of substantial scientific interest due to their unusual chemical and physical properties. In a seminal study on thermoelectric materials [1], it was predicted that dimensional reduction of matter, such as a quantum-well superlattice structure, can significantly increase the figure-of-merit (ZT) value. According to calculations for Bi_2_Te_3_, the ZT value can be increased by a factor of thirteen in comparison with the bulk value [1]. Despite the fact that low-dimensional particles (or quantum dots, superlattices, etc.) have, in general, the same atomic structure as their parent micro-sized material, they must be considered as a new material with its own unique properties [2]. Nowadays, many thermoelectric materials are being explored for power generation applications, such as GeTe [3], PbTe [4], half-Heusler [5], and skutterudites [6]. The current interest in PbTe nano-bars arises from their useful thermoelectric properties [7]. It is known that the presence of nano-bar-like formations in spark-plasma sintered (SPS) PbTe specimens increases the ZT value to approximately 0.45 at 300 K. Additionally, the total thermal conductivity is reduced [4,8]. Further studies reported that Se-doped and Tl-doped PbTe materials containing nano-bar-like formations of the microstructure reveal very attractive thermoelectric properties [1,9]. All considerations that focus on improving the ZT value of PbTe may only have a substantial impact if an appropriate synthesis method can be found.

One of the alternative ways to prepare PbTe nanoparticles free of surface contaminations is the electron-beam treatment. Electron-beam interaction with a material is governed by the principles of energy and momentum conservation [10]. During the interaction, electrons transmit some energy to the material through collisions. If the injected energy is large enough, atoms can be permanently displaced from their prior positions. Particle fragmentation occurs when the current density reaches a threshold value at which evaporation of the sample sets in. The threshold can be reached by gradually converging the electron-beam diameter. Therefore, the transmission electron microscope (TEM) cannot only act as observation equipment, but it can also be used as a model setup for manufacturing processes at the nanoscale.

In this paper, we investigate recrystallization at the nanoscale of powdered single-crystal and polycrystalline PbTe under electron-beam treatment resulting in the formation of nanoparticles with a parallelepiped-like shape (nano-bars) and extended, two-dimensional nm-sized architectures (nano-layers). The PbTe single crystal was prepared with an excess of tellurium [11]. The first TEM investigations of the lead telluride single crystal showed that in the tellurium-rich, as-grown sample, small and homogeneously distributed tellurium precipitates with defined lattice orientation were present. Additionally, besides low-angle grain boundaries and dislocations, Moiré patterns and needle-like precipitations were also observed. The estimation of the number of Te atoms absorbed by the precipitates corresponds to the maximum deviation from stoichiometry, and supports the idea of diffusion-determined precipitation due to the retrograde course of the solidus line, which causes a supersaturation of the excess component and results in precipitates within the crystals being cooled after growth [12]. A general method and the details of the single-crystal growth are described in References [13,14], see also the Materials and Methods section.

## 2. Materials and Methods

### 2.1. Crystal Growth and Preparation of TEM Specimens

Polycrystalline PbTe was synthesized by melting elements (Pb—99.999 mass %, Te—99.9999 mass %) at 1273 K for 6 h under vacuum (~10^4^ Torr). The product was ground in an argon atmosphere by mortar and pestle (both from agate) and then densified by spark-plasma sintering (SPS, Fuji, SPS-515S, Saitama, Japan) at 673 K and a pressure of 60 MPa. 

The PbTe single crystal was grown using the vertical Bridgman–Stockbarger method [13]. The melt composition was chosen to be near stoichiometric, with a slight excess of Te (0.5%) [14]. To prepare the TEM specimens from the polycrystalline sintered ingot and the single crystal, the materials were ground by mortar and pestle for several minutes with a minimum applied force. Subsequently, the powder was mixed with methanol, and the obtained suspension was transferred to the holy-carbon TEM grid.

### 2.2. TEM

Transmission electron microscopy investigations were performed using a FEI Tecnai F30-G2 equipped with a field-emission gun and Super-Twin lens (FEI Eindhoven, NL, USA) at an acceleration voltage of 300 kV. The microscope is equipped with a wide-angle slow-scan CCD camera (MultiScan, 2×2 k pixels; Gatan Inc., Pleasanton, CA, USA), possesses a point resolution of 2.0 Å, and the information limit is about 1.2 Å. Spherical aberration corrected, high-resolution TEM analyses of the sample were performed with a JEM-ARM300F microscope (Grand ARM, JEOL, Akishima, Japan) with double correction. Dodecapole correctors in the beam and the image forming system corrected the spherical aberration of the condenser and the objective lenses, respectively. The TEM resolution was 0.5–0.7 Å, depending on the resolution criterion applied. TEM images were recorded on a 4 k × 4 k pixel CCD array (Gatan US4000). The data sets generated or analyzed in the current study are available from the corresponding author upon request.

In order to generate nano-bars and nano-cubes in the TEM by PbTe depletion, an acceleration voltage of 300 kV was applied. The dose rates for recording images and videos typically amounted to 10.000–15.000 electrons/nm^2^ s. The irradiated area of interest was about 10–20 μm^2^ at a magnification of about 20.000×. The irradiation time needed for the formation of nano-bars and nano-cubes amounted to about 1–2 min, see Supplementary Videos S1 and S2. In order to follow the depletion mechanism of PbTe at an atomic resolution a much larger dose was required. At magnifications from about 520.000× up to 646.000×, the applied dose amounted to ~2.600.000 e/nm^2^ s and 16.000.000 e/nm^2^ s. The time period for the observation of atomistic depletion amounted to about 22 min.

## 3. Results

### 3.1. Nano-Recrystallization of Powdered Single Crystal of PbTe under Electron-Beam Conditions

**Figure 1:**
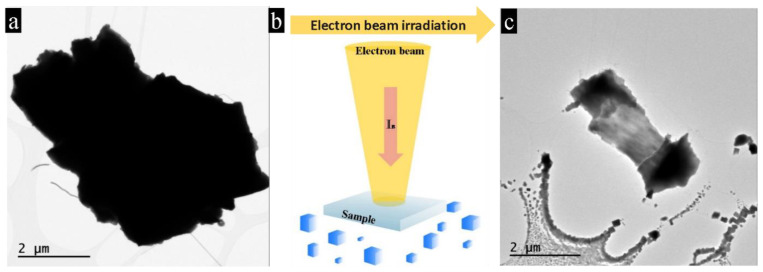
Formation of PbTe nano-bars during the electron-beam treatment. (**a**) Initial PbTe particle from an as-prepared single crystal before heavy irradiation. (**b**) Scheme of the formation of PbTe nano-bars. (**c**) The same PbTe particle after beam irradiation is remarkably decreased in size and is surrounded by nanoparticles of different sizes produced by the electron beam at bottom of the micrograph.

The fragmentation of PbTe micro-particles was clearly observed after electron-beam treatment beyond a certain threshold value of the current (Figure 1). The threshold current was in the range of 10.000–15.000 e/nm s., for details see the Materials and Methods section. When the initial single-crystalline PbTe particle (Figure 1a) was exposed to electron irradiation, it partially evaporated, see scheme Figure 1b. The evaporated material transferred to the colder part of the carbon substrate and produced rectangular-shaped particles (hereafter called nano-bars) measuring 4–100 nm (Figure 1c and Supplementary Video S1). The sizes and number of nano-bars decreased with increasing distance from the initial evaporated particle. Close to the primary particle, nanoparticles were abundant and mostly interconnected. The regions far apart of the primary particle showed only tiny and isolated nano-bars (Figure 1c).

**Figure 2:**
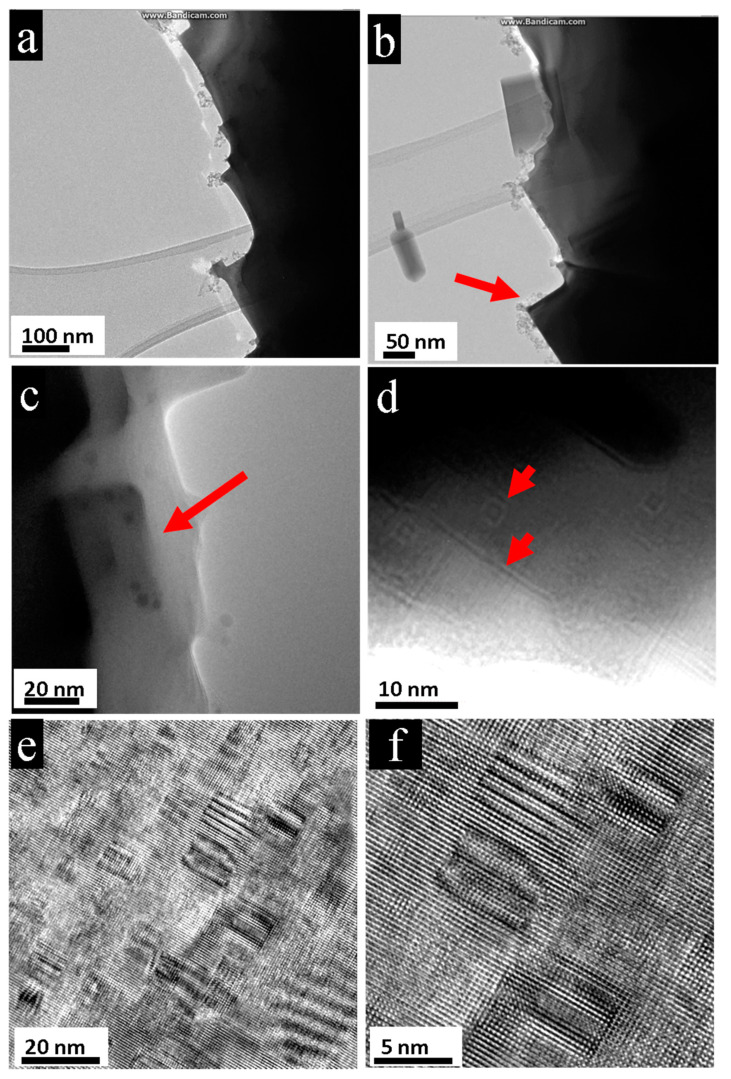
Nanostructuring of PbTe crystal before and after electron-beam irradiation. (**a**,**b**) During and after irradiation, the crystal shows clear geometric rectangular boundaries. (**c**,**d**) Transparent edges of the irradiated aggregate where the shapes of nano-cubes become evident, follow red arrows. (**c**) Large cube of about 40 nm. (**d**) Smaller and nested cubes from 2 to 8 nm. (**e**,**f**) The initial crystal exhibits clear nanopatterning: nano-cubes ranging mainly from 5 to 15 nm.

The initial crystals as well as the depleted aggregates showed nanostructuring with distinct nano-bars or nano-cube patterns. During and also after irradiation, the expended crystal displayed linear and rectangular boundaries, see Figure 2a,b. Magnified images of the transparent edges revealed the inside of the sample nano-bars and nano-cubes of different sizes. Besides the large cubes (about 40 nm in size (Figure 2c)), nested patterns of smaller cubes could also be imaged (Figure 2d). The same nanopatterning appeared on the initial single crystal before irradiation, when nested 5–15 nm nano-cubes were registered in the high-resolution images, see Figure 2e,f.

**Figure 3:**
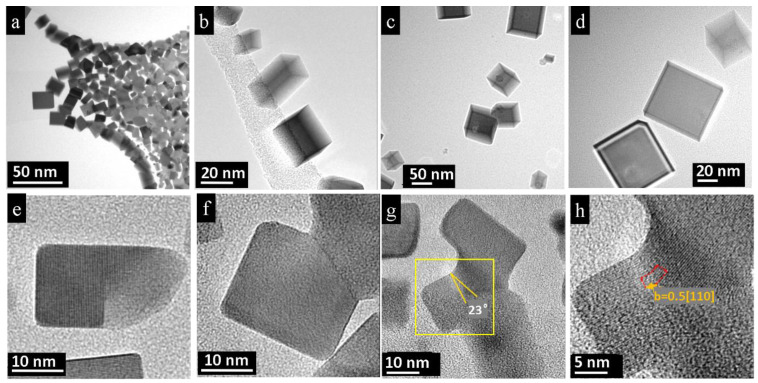
Electron-beam treatment products of the PbTe single crystal: (**a**) Overview of interconnected PbTe nano-bars. Near the initial crystal, a large amount and high density of cubes and bars occur, which form a 2D layer. (**b**–**d**) At larger distances away from the initial aggregate, the number of nanoparticles drops; thus, individual and well-separated nano-bars with different orientations and aspect ratios can be observed. (**e**) HRTEM image of a partially formed nano-bar. (**f**) Neighboring nano-bars with imperfect attachment. (**g**,**h**) Fused cubes with edge dislocation, Burgers vector *b* = *a*/2⟨110⟩.

The irradiation products also consisted of nano-bars and nano-cubes, see Figure 3a–d. In the vicinity of the initial crystal, we find a high density of nano-bars and nano-cubes mostly in [100] orientation, giving rise to a continuous, thin film, see Figure 3a. At larger distances, local particle density decreases, and isolated individuals of nanoparticles appeared, see Figure 3b–d. They possessed different shapes, and some of the nano-bars showed step-like edges (Figure 3e). Such unfinished bars are rare, i.e., the rectangular shape is preferably completed during the growth process. The resulting nanoparticles were stable upon exposure to the electron beam and were not fragmented by further treatment. Some bars and cubes merged by contact even though the initial orientation remained different. The growing particles were randomly interconnected; therefore, one observes perfect nano-bars with imperfect attachments (Figure 3f–h). The imperfect attachments of the nanoparticles usually result in edge dislocations [15,16]. Indeed, frequently the particles attached on {100} facets, and edge dislocations with Burgers vector *b* = *a*/2⟨110⟩ were observed (Figure 3g,h). Such a dislocation has the lowest energy and is the most preferable [15]. Such a mismatch is one of the reasons why some cubes are not absorbed by others.

**Figure 4:**
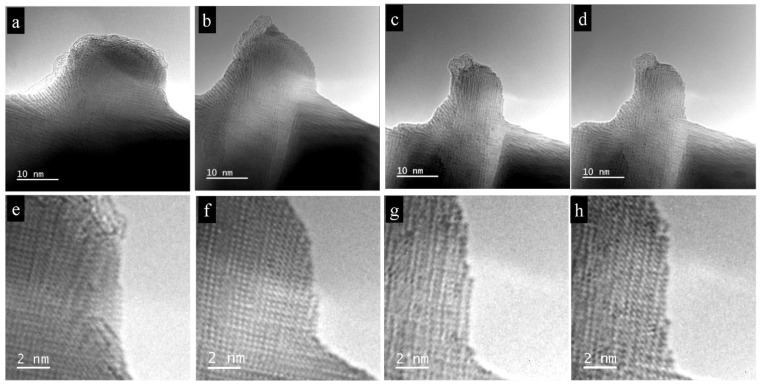
Depletion of the initial single-crystal particle. (**a**–**d**) Depletion of an integrated nano-bar-like formation along the edge of the initial crystal, leading to a buckling at the final state. The size decreases continuously from 30 nm to about 10 nm. At the top of the bar, curling and fibrillation occurs. (**e**–**h**) Layer-by-layer depletion of the initial PbTe particle.

The irradiation products consisted of nano-bars and nano-cubes-like the initial single crystal particle; consequently, the idea of a depletion mechanism, whereby these cubes are set free from the initial single crystal, is suggested. Therefore, we decided to perform in situ experiments to confirm the mechanism of decomposition by the initial nanostructuring of the single crystal. Figure 4a–d shows the in situ depletion experiment on a cube-like formation along the edge of the initial single crystal. The depletion led to a diminution in diameter of the rectangle, from 30 nm to about 10 nm, and to deformations and tension within the PbTe lattice. At the top, curling and fibrillation occurred, possibly due to dissolution and the splitting-off of the PbTe crystal structure into amorphous substructures during the evaporation process. In situ investigations show that atoms evaporated from their initial positions through atomic-column-by-atomic-column or layer-by-layer processes. Therefore, the electronic-beam treatment did not produce vacancies inside the initial particles (Figure 4e–h). This observation disproves the assumption of depletion and disintegration of the initial PbTe aggregate by inherent existing nano-cubes and bars, but it shows an atom-by-atom depletion mechanism.

**Figure 5:**
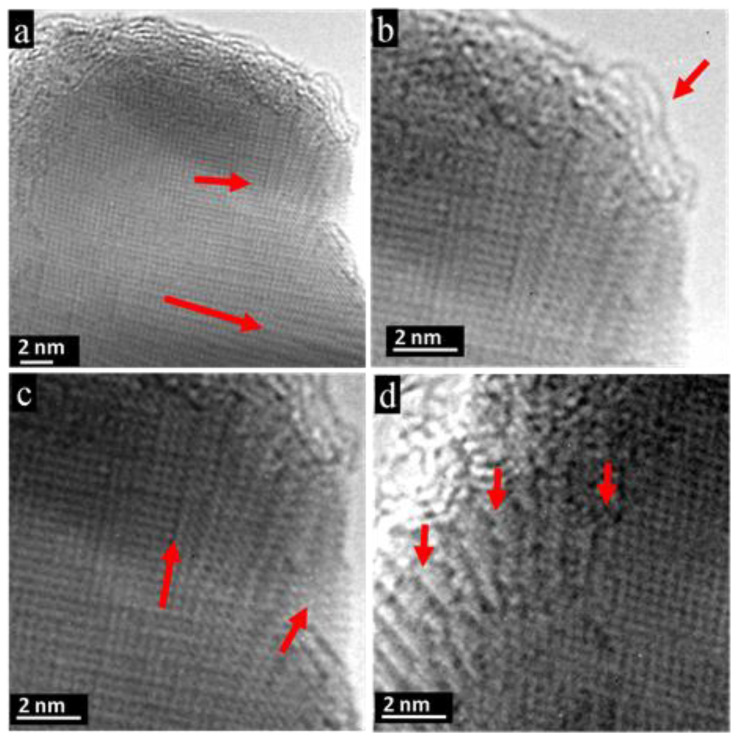
Depletion mechanism of PbTe. (**a**) Stress formation on PbTe crystal during depletion causes divergence of about 5 degrees between convexity and matrix concerning the (100) direction, see red arrows. (**b**) At the top of the convexity, atomic rows seem to be split from the uppermost surface, giving rise to fibrillation and coiling. (**c**) In addition, the (010) direction shows a divergence of about 10 degrees caused by internal tension. (**d**) Degradation of crystal lattice occurs layer-wise and atom-by-atom, as visualized at the edge of the convexity. Several typical kinks and steps are marked by red arrows.

Further magnification of the snapshot in Figure 4a, recorded during the depletion process, revealed high tension and stress within the sample caused by the high-energy input. The 100 direction within the cube-like convexity (upper red arrow) differed by about 5 degrees from the 100 direction within the matrix below (red arrow at bottom), see Figure 5a. At the top, the outermost atomic rows of the crystal seemed to detach from the surface and tended to form fibril-like structures, see Figure 5b, red arrow. In addition, within the convexity, diversification of the lattice planes occurred (Figure 5c), where the 010 direction showed 10 degrees’ divergence from the inner part (red arrow, left) when moving to the edge (red arrow, right). The layer-by-layer removal of PbTe is nicely seen on the left edge of the convexity, and steps and kinks on the atomic level are clearly pictured, follow e.g., red arrows (Figure 5d).

**Figure 6:**
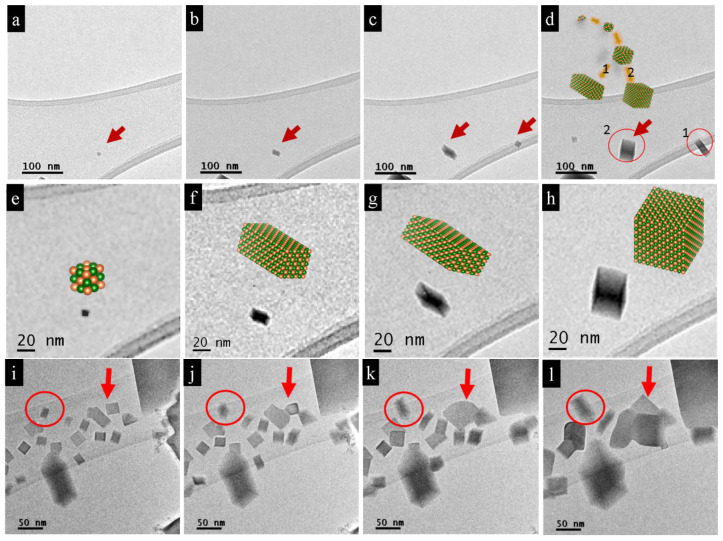
Formation of PbTe nano-cubes and nano-bars during the electron-beam treatment. (**a**–**d**). Overview of images of a single nano-bar nucleation on the carbon substrate during the melting of the initial µm-sized particles. (**a**) Initial and (**b**–**d**) subsequent gradual growth of nucleated PbTe nano-bars. (**e**–**h**) Magnified micrographs and snapshots of the growth process of the pitched-on particle marked with a red arrow in (**a**). (**e**) The starting size is about 8 nm when the particle could be clearly imaged and (**h**) about 40 nm in the final state of the observation period. The particle undergoes different aspect ratios being, e.g., brick-like (**g**) to a subsequent cube-like (**h**) form. (**i**–**l**) Growth of an assembly of nano-bars, one dominant rod marked by a red arrow grows incorporating fast vicinal particles. Nano-bar marked with a red circle develops freely without interfering with other nanoparticles.

The first step of the nano-bar formation is nucleation, which is subsequent to sublimation, where the formation of bars takes place at different times and is indicative of a stochastic process (Figure 6a–d and Supplementary Video S2) [17]. The distinguishing feature is that all nano-bars, even the smallest (around 4 nm) ones, possessed a rectangular shape. PbTe nanoparticles form in this geometry due to free-energy minimization [18]. However, the growth of isolated nanoparticles after reaching a certain cube size of 10–20 nm follows two possible patterns (Figure 6d). The first possibility is that the particle predominantly increases in size along one direction while growing only gradually in the other two directions (marked as 1 in Figure 6d). This type of growth behavior was observed at the edges of the carbon film near the vacuum. The second growth pattern, in which all directions develop equally, was observed for particles located on the larger continuous carbon film areas (marked as 2 in Figure 6d). Figure 6e–h shows enlarged snapshots of the growth mechanism of the nanoparticle marked with a red arrow in 6a. Commonly, coalescence events were observed between nanoparticles (Figure 6i–l). Nano-bars, which nucleated at a short distance between them, may come into contact with each other after growing and coalescing, see e.g., assembly in Figure 6l marked with a red arrow. Larger particles incorporated smaller ones on the principle of gradual rebuilding of the crystal structure, i.e., smaller bars are used as a supplementary building material for larger particles. This behavior is known as the Ostwald ripening mechanism. Its driving force is the decrease in surface free energy [19]. The coalescence may develop also in another manner, namely when small particles fuse together and become larger. Because of the crystallographic directional mismatch, attached particles continued to grow further in their own directions. This occurs because the growth direction depends on the orientation of the primary nucleus on the carbon film.

### 3.2. Nano-Recrystallization of Spark-Plasma Sintered Polycrystalline PbTe

Annealing of polycrystalline PbTe at 400 °C causes a change from a metal-semiconductor into an intrinsic semiconductor [20]. Therefore, post-annealed polycrystalline PbTe should possess a more ordered crystal structure.

**Figure 7:**
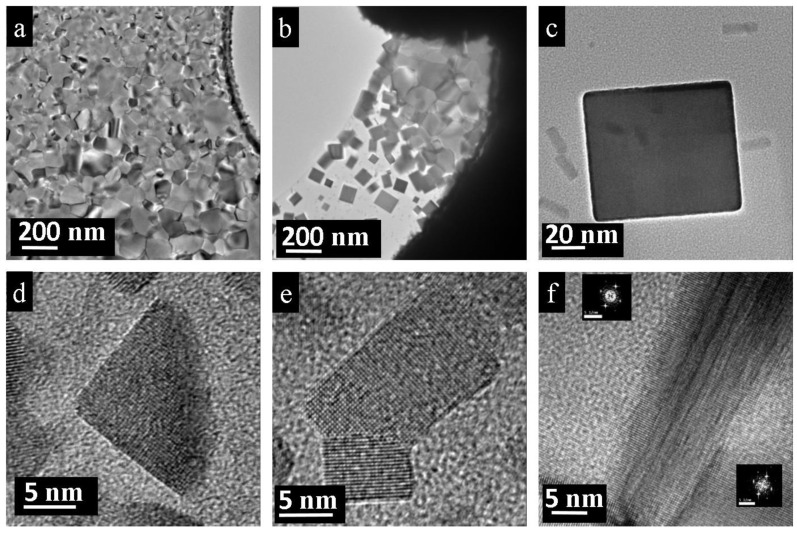
Electron-beam treatment of polycrystalline PbTe annealed at 400 °C: (**a**) Overview of a layer. (**b**) Formation of the large nano-bars next to the source particle. (**c**) Individual, very large nano-bar. (**d**) Partially formed nano-bar. (**e**) Imperfect attachment of nanoparticles with a screw dislocation. (**f**) Edge dislocation on the border between two grains both oriented in the [100] direction.

The mechanism of nano-bar formation from polycrystalline PbTe is analogous to the single-crystal situation. Compared to the single-crystal material, however, fragmentation of poly-crystalline PbTe requires higher beam current densities. Even under harsher conditions, particles did not decompose completely. After beam treatment, the adjacent area around the source particle was covered by a dense layer of PbTe nano-bars (Figure 7a). The thickness of the layer was below 100 nm, concluded from the fact that grains were transparent for electrons. The closer the investigated film areas are to the source particle, the denser and larger the grains are. Grains close to the center were approximately 100–500 nm and had random orientation. They did not contain precipitations or dislocations. Occasionally, grains with the [100] orientation were attached to each other (Figure 7f). A fast Fourier transform reveals that these grains were imperfectly attached to each other and contained dislocations at the grain boundary. Further away from the initial particle, the layer became less dense, and at a certain distance only isolated nano-bars were observed (Figure 7b). The treatment of a 10 µm-sized particle produced sometimes large nano-bars of approximately 100 nm with free areas between the nanofilm and the source particle (Figure 7c), i.e., the recrystallization in these cases was fast and complete. Because this process requires a constant source of initial material, such nano-bars only appear close to the source particle and, consequently, have a perfect shape and structure. We did not observe any dislocations or vacancies within the formed large nano-bars. The vast majority of the bars were partially or completely interconnected with each other.

On the periphery, the layer dissipated and transformed into partially interconnected or individual nano-bars of different sizes (Figure 7e). Essentially, partially interconnected nano-bars are imperfectly attached to each other, and, as a consequence, these attachments result in edge dislocations. Moreover, the particles may even be twisted, relative to each other. Such a twist around the attachment region produces a screw dislocation and introduces a slight contrast difference because the upper nano-bar is rotated off of the [100] zone (Figure 7e). On the other hand, the separated nano-bars at the periphery exhibited a perfect structure and were free of point defects or precipitations (Figure 7d). Even the smallest particles (<4 nm) remained rectangular in shape.

**Figure 8:**
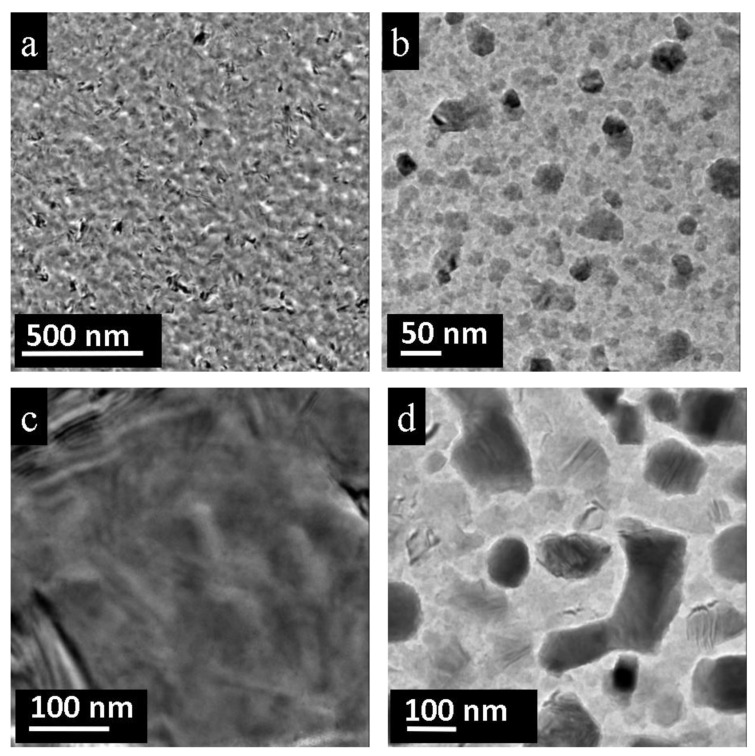
PbTe layers obtained by electron-beam treatment of the polycrystalline PbTe (pre-annealed at 400 °C) after additional heat treatment at 350 °C (1 h)**.** (**a**,**b**) Layers formed at a larger distance from the initial particle: (**a**) Overview of a layer composed of fine grains forming a dense packing with some dislocations within the layer. (**b**) In addition, less dense and more inhomogeneous covering occurs revealing individual isolated grains and roundish nano-particles. (**c**,**d**) Layers formed very near to the initial particle: (**c**) Homogenous film is observed in direct vicinity of the initial crystal. (**d**) At the edges, layers composed of isolated, large nano-bars occur near the source particle.

However, the formed layer was not perfect and contained holes and grain boundaries. In order to improve the PbTe layer quality, the grid with nano-particles was additionally annealed at 350 °C for one hour. The relatively low temperature (PbTe forms congruently at 924 °C [14]) was chosen because previous annealing experiments showed that bulk PbTe is subject to grain coalescence if annealed at 400 °C [20]. Different scenarios of layer morphology and homogeneity were detected depending on the distance from the initial crystal treated by the electron beam. With increasing distance from the center, the structure of the layer altered from homogeneous to irregular with some holes and precipitations (Figure 8a). The further the distance from the source particle, the more dislocations and imperfections in the layer occur. A similar situation was observed at the layer periphery. After heat treatment, all particles lost their initial rectangular shapes and assumed more rounded shapes (Figure 8b). On the contrary, in the neighborhood of the initial crystal, a different layer morphology appeared. In the region shown in Figure 8c, after one hour of annealing nearly all grain boundaries dissipated. Instead, a uniform PbTe layer with an abundant number of dislocations was observed (Figure 8c). At the same time, giant nano-bars next to the center were increasingly destroyed and lost their perfect shape (Figure 8d). The free space between particles is filled with melted product.

## 4. Discussion

**Figure 9:**
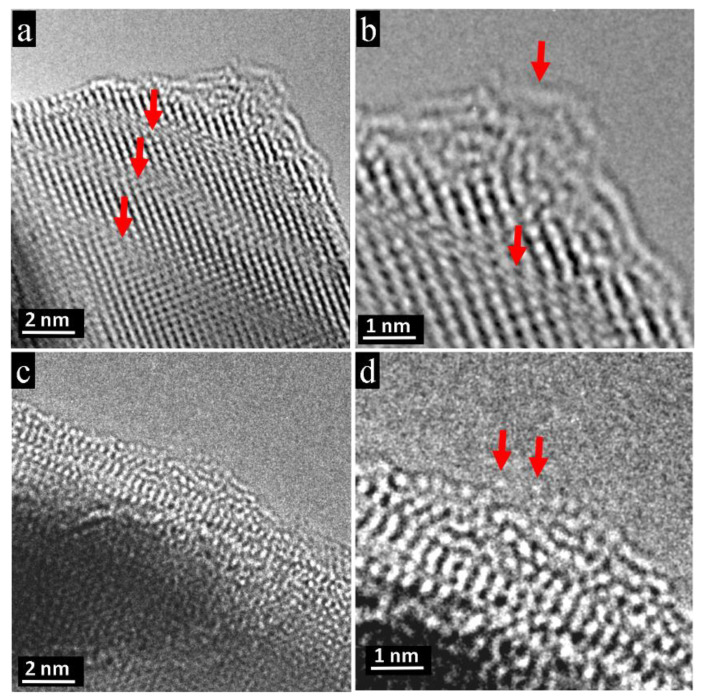
Degradation mechanism at atomic level revealed on outermost PbTe atomic layers obtained by electron-beam treatment of the single crystalline PbTe. (**a**) Degradation is manifested by atomic steps, in this case along the [110] direction, showing layer-wise depletion, see red arrows. (**b**) The corners are the most energetically unstable places where disintegration of atomic rows occurs. (**c**) Corrugated uppermost atomic layers. (**d**) Magnification reveals the atomistic degradation mechanism. An atom is removed from the very top from the necklace-like atomic row, leaving two open ends of atomic chains marked by red arrows.

During electron-beam irradiation, the fragmentation of the initial particles happens via a sequential removal of the outermost atomic layers from the surface, see Figure 4e–h, Figure 5d, and Supplementary Video S2 of in situ experiments. The PbTe crystal lattice on the very surface becomes mobile and tends to form disintegrated atomic layers, see Figure 9. In Figure 9a, atomic steps are pictured and marked with red arrows together with a disordered top layer. The magnification in Figure 9b displays the fibrillated and bent atomic layers. The disordered uppermost layer can also be visualized atom-by-atom, as demonstrated in Figure 9c, when sample motion can be ruled out. Enlargement of Figure 9d reveals the break at the top of the necklace-like layer into two parts. The gap between the ends of the atomic chains marked by red arrows marks the removal of one atom (lead or tellurium).

Removed material sublimes, gradually forming new nanostructures in the adjacent area of the carbon film. As a result of the limited area around newly nucleated particles, they join together and coalesce. Commonly, the larger particles absorb the smaller ones. However, nanoparticles which are imperfectly attached at {100} facets did not coalesce. Their attachment results in a dislocated formation, and each continues to grow further in its own direction. All isolated particles exhibit rectangular shapes. The smallest nano-bars are smaller than 4 nm, and the largest may grow up to 100 nm.

The particles of polycrystalline PbTe (initially annealed at 400 °C) mostly decompose if exposed to the electron beam and form a nanolayer within an area of 30 µm around the initial particle. This nanolayer is composed of PbTe grains of different sizes, which do not have dislocations or impurities. The nanolayer is not uniform and occasionally disintegrates into larger nano-bars close to the initial particle and smaller nano-bars at the layer periphery. The additional annealing of the nanolayer at 350 °C for one hour yields a uniform, homogenous, and grain-boundary-free layer with dislocations.

Nanostructuring through the presence of nested formation of bars and cubes within the initial crystal under investigation suggested that preformed nano-bars within the bulk crystal are set free by the electron beam. Nanostructuring often occurs in doped samples and is responsible for the superior performance of thermoelectrics [21,22]. However, in our case, doping was not performed; thus, only self-doping given by a slight excess of tellurium can be considered as a reason for this intrinsic nanostructuring of our initial single crystal. Nevertheless, our in situ depletion experiments show that the nano-bars, generated outside the initial crystal, are formed atom-by-atom from the gas phase. The degradation of the initial crystal occurs atom-by-atom and creates a suitable gas atmosphere for the formation of nanoaggregates. Former investigations of chemical transport reactions stated that thermal depletion of PbTe results in mono-atomic Pb and Te, in addition to dimeric Te_2_ and monomeric PbTe species, in the vapor phase [23].

## 5. Conclusions

The decomposition of initial PbTe particles under well-defined electron-beam conditions (threshold value of current) may be understood by the crystal structure imperfections inherent to lead telluride at temperatures below 450 °C. The atomistic depletion mechanism could be revealed at the atomic level by high-resolution TEM, proving that degradation occurs atom-by-atom, evoking internal stress and fibrillation at the very surface. The formation of nano-bars and nano-cubes could be followed up in situ. The recrystallized nanoparticles in turn exhibit an “ideal” crystal structure, which may explain their relative stability with respect to the beam in comparison with the initial particles. Recrystallization by electron-beam treatment is an alternative way to nanostructure PbTe. Direct formation of nano-bars and nano-layers of lead telluride using electron-beam treatment is a particularly interesting technique because it produces nanostructures free of synthesis-educts contamination.

## Data Availability

The data presented in this study are available on request from the corresponding author.

## Supplementary Materials

The following are available online at https://www.mdpi.com/2079-4991/11/1/163/s1, Video S1: depletion crystal, Video S1: nanobarn formation.
